# Enhanced Production of Pterostilbene in *Escherichia coli* Through Directed Evolution and Host Strain Engineering

**DOI:** 10.3389/fmicb.2021.710405

**Published:** 2021-10-07

**Authors:** Zhi-Bo Yan, Jing-Long Liang, Fu-Xing Niu, Yu-Ping Shen, Jian-Zhong Liu

**Affiliations:** ^1^Institute of Synthetic Biology, Biomedical Center, Guangdong Province Key Laboratory of Improved Variety Reproduction in Aquatic Economic Animals, School of Life Sciences, Sun Yat-sen University, Guangzhou, China; ^2^College of Light Industry and Food Science, Zhongkai University of Agriculture and Engineering, Guangzhou, China

**Keywords:** pterostilbene, *de novo* biosynthesis, directed evolution, genome engineering, *Escherichia coli*

## Abstract

Pterostilbene is a derivative of resveratrol with a higher bioavailability and biological activity, which shows antioxidant, anti-inflammatory, antitumor, and antiaging activities. Here, directed evolution and host strain engineering were used to improve the production of pterostilbene in *Escherichia coli*. First, the heterologous biosynthetic pathway enzymes of pterostilbene, including tyrosine ammonia lyase, *p*-coumarate: CoA ligase, stilbene synthase, and resveratrol O-methyltransferase, were successively directly evolved through error-prone polymerase chain reaction (PCR). Four mutant enzymes with higher activities of *in vivo* and *in vitro* were obtained. The directed evolution of the pathway enzymes increased the pterostilbene production by 13.7-fold. Then, a biosensor-guided genome shuffling strategy was used to improve the availability of the precursor L-tyrosine of the host strain *E. coli* TYR-30 used for the production of pterostilbene. A shuffled *E. coli* strain with higher L-tyrosine production was obtained. The shuffled strain harboring the evolved pathway produced 80.04 ± 5.58 mg/l pterostilbene, which is about 2.3-fold the highest titer reported in literatures.

## Introduction

Stilbenes are a small category of the secondary metabolites of plants derived from the general phenylpropanoid pathway. Naturally occurring stilbenes are found primarily in grapes, peanuts, blueberries, and some medical plants (Tsai et al., 2017). Stilbenes play a major role in the defense response of plants; thus, their abundance is expected to increase when the plants are exposed to some harsh environmental conditions, such as UV irradiation and fungal infection (Wang et al., 2010). Numerous scientific studies have revealed the broad range of health-improving activities of stilbenes including antioxidant activity, anti-inflammatory activity, antitumor activity, and antiaging activity (Burns et al., 2002; Ulrich et al., 2005; Baur et al., 2006; Campagna and Rivas, 2010). Pterostilbene is a structural analogy of the well-studied stilbene resveratrol. Because of the substitution of hydroxyl with methoxy groups, the lipophilicity of pterostilbene is enhanced substantially, enhancing the increased bioavailability and biological activity of pterostilbene in comparison with that of resveratrol (Shao et al., 2010; Kosuru et al., 2016).

Owing to the diverse health-promoting properties of stilbenes, heterologous biosynthesis of stilbenes using the engineered microbial cell factories has been under the spotlight of the research field of metabolic engineering in recent years. Some groups have successfully engineered *Saccharomyces cerevisiae* (Jeandet et al., 2012; Li M. et al., 2015; Li et al., 2016), *Escherichia coli* (Camacho-Zaragoza et al., 2016; Liu et al., 2016; Wu et al., 2017; Hong et al., 2020), and *Yarrowia lipolytica* (He et al., 2020; Saez-Saez et al., 2020) for the production of resveratrol. However, only two papers reported the *de novo* production of pterostilbene by using engineered microorganisms. The *de novo* biosynthesis of pterostilbene from glucose was achieved for the first time by the introduction of the methyltransferase from *Vitis vinifera* into resveratrol-producing *S. cerevisiae* (Li et al., 2016). The engineered *S. cerevisiae* produced 34.93 mg/l of pterostilbene from glucose (Li et al., 2016). Heo et al. (2017) also engineered *E. coli* for the *de novo* biosynthesis of pterostilbene from glucose by the overexpression of caffeic acid O-methyltransferase from *Arabidopsis thaliana*, tyrosine ammonia lyase (TAL) from *Saccharothrix espanaensis*, *p*-coumarate: CoA ligase from *Nicotiana tabacum*, and stilbene synthase (STS) from *V. vinifera* in the L-Tyr-overproducing *E. coli* strain. The engineered *E. coli* produced 33.6 mg/l of pterostilbene from glucose in shake flask cultures. The titer of pterostilbene is much lower than that of resveratrol (12.4 g/l in *Y. lipolytica*, 812 mg/l in *S. cerevisiae*, and 304.5 mg/l in *E. coli*) (Li et al., 2016; Wu et al., 2017; Saez-Saez et al., 2020). Therefore, it is of great necessity to build a biotechnological platform for the biosynthesis of pterostilbene.

**FIGURE 1:**
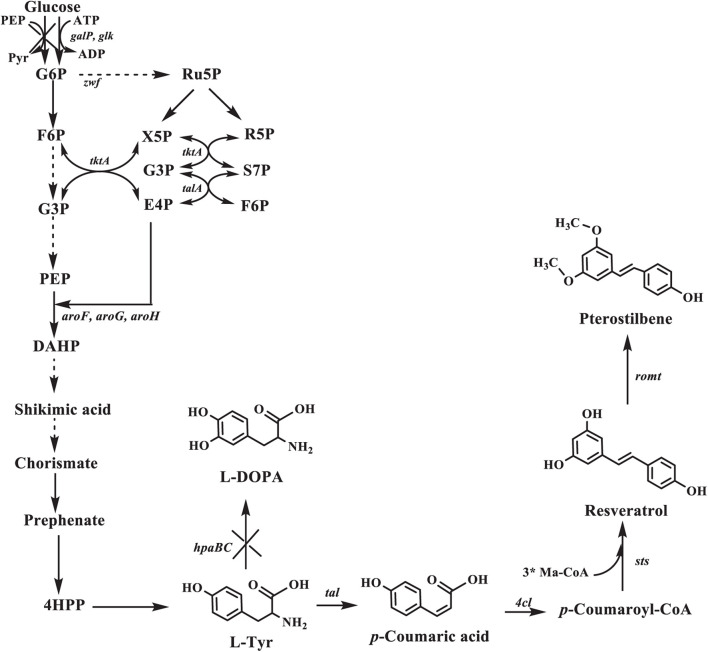
Pterostilbene biosynthetic pathway in *Escherichia coli*. The ×’s indicate that the gene is deleted. G6P, glucose 6-phosphate; F6P, fructose-6-phosphate; G3P, glyceraldehde-3-phosphate; Ru5P, ribulose-5-phosphate; X5P: xylulose-5-phosphate; R5P, ribose-5-phosphate; S7P, sedoheptulose-7-phosphate; PEP, phosphoenolpyruvate; E4P, D-erythrose-4-phosphate; DAHP, 3-deoxy-D-arabino-heptulosonate-7-phosphate; L-Tyr, L-tyrosine; L-DOPA: L-3,4-dihydroxyphenylalanine; *galP*, galactose permease gene; *glk*, glucokinase gene; *zwf*, glucose-6-phosphate dehydrogenase gene; *tktA*, transketolase 1 gene; *talA*, transaldolase A gene; *aroF*, *aroG*, *aroH*, DAHP synthase gene; *hpaBC*, p-hydroxyphenylacetate 3-hydroxylase gene; *tal*, tyrosine ammonia-lyase gene; *4cl*, 4-coumarate:CoA ligase gene; *sts*, stilbene synthase gene; *romt*, resveratrol O-methyltransferase gene.

In this study, we created a biosynthetic pathway of pterostilbene in an L-tyrosine producer for the *de novo* pterostilbene biosynthesis. Pterostilbene biosynthesis starts from the endogenous L-tyrosine (L-Tyr) and involves the four enzymes (Figure 1). In this pathway, L-tyrosine is converted into *p*-coumaric acid using TAL. p-Coumaric acid is then activated to p-coumaroyl-CoA with the *p*-coumarate CoA ligase (4CL). This p-coumaroyl-CoA is condensed with three molecules of malonyl-CoA *via* STS. Resveratrol is converted into pterostilbene by resveratrol O-methyltransferase (ROMT). First, the four enzymes were directly evolved to increase the pathway efficiency.

In our previous study (Niu et al., 2020b), we developed a strain improvement strategy of biosensor-guided genome shuffling and applied this strategy to improve the production of shikimic acid in *E. coli*. In this method, a biosensor was used for screening the mutant with improved phenotype from the shuffled library after genome shuffling. Thus, a biosensor-guided genome shuffling strategy was then used to construct a host strain for increasing the precursor L-Tyr availability. Finally, introducing the evolved pathway into the L-Tyr hyper-producer resulted in the production of pterostilbene to 80.04 ± 5.58 mg/l.

## Materials and Methods

### Strains, Plasmids, and Primers

The bacterial strains and plasmids used in this study are listed in Table 1. The primers used in this study are presented in Supplementary Table 1. *E. coli* DH5α was used for the construction and propagation of plasmids. L-tyrosine producer *E. coli* TYR-30 (Wei et al., 2016) was used as the starting host strain for the production of pterostilbene.

**TABLE 1:** Strains and plasmids used in this study.

Name	Description	References/source
**STRAIN**		
*Escherichia coli* DH5α	*supE44* Δ*(lacZYA-argF) U169* (Φ*80lacZ* Δ*M15*) *hsdR17 recA endA1 gyrA96 thi-1 relA1*	Invitrogen
*E. coli* BW25113	*lacI^*q*^ rrnB_*T*__14_*Δ*lacZ_*WJ*__16_ hsdR514*Δ*araBAD_*AH*__33_*Δ*rhaBAD_*LD*__78_*	Datsenko and Wanner, 2000
*E. coli* DOPA-30N	L-DOPA overproducer	Wei et al., 2016
*E. coli* TYR-2-7H3	L-tyrosine overproducer, obtained from ARTP mutagenesis of *E. coli* TYR-30-RAG	This study
*E. coli* TYR-14B1	L-tyrosine overproducer, obtained from genomic shuffling of *E. coli* TYR-2-7H3 by ep-WGS	This study
*E. coli* DOPA-30	L-DOPA overproducer	Wei et al., 2016
*E. coli* TYR-30	L-tyrosine producing strain, *E. coli* DOPA-30, Δ *hpaBC*	This study
*E. coli* TYR-30-RAG	L-tyrosine overproducer, *E. coli* TYR-30 derivative deleted the 2-acyl-glycerophosphoethanolamine cycle	This study
**PLASMID**		
pZAC	Constitute expression vector, p15A ori, P37 promoter, Cmr, BglBrick, ePathBrick containing four isocaudamer (*Avr*II, *Nhe*I, *Spe*I, and *Xba*I)	Li X. R. et al., 2015
pZAC-tal-4cl	pZAC derivative, carrying the gene cluster of *tal* from *R. glutinis* and *4cl* from *A. thaliana*	This study
p214C	pZAC-tal-4cl derivative, with the gene cluster of *tal* from *R. glutinis* and *4cl* from *A. thaliana* directly co-evolved	This study
pZBK	BglBrick/ePathBrick expression vector, pBBR1 *ori*, P37 promoter, Kan^r^	Li X. R. et al., 2015
pZBK-sts	pZBK derivative, carrying the *sts* gene from *Vitis vinifera*	This study
pZBK-sts53	pZBK-sts derivative, with the *sts* gene from *V. vinifera* directly evolved	This study
pZBK-romt	pZBK derivative, carrying the *romt* gene from *V. vinifera*	This study
pZBK-romt18	pZBK-romt derivative, with the *romt* gene from *V. vinifera* directly evolved	This study
pZBK-sts-romt	pZBK derivative, carrying the gene cluster of *rs* and *romt* from *V. vinifera*	This study
pZBK-sts53-romt18	pZBK derivative, constructed by reassembly of the separately directly evolved *sts* and *romt* from *V. vinifera*	This study
pTtgR	Resveratrol biosensor	Xiong et al., 2017
pSen-tyr	L-tyrosine biosensor	This study
pCas*	*E. coli* cas9 (K848A/K1003A/R1060A) expression vector	Niu et al., 2018
pTargetB	E. coli sgRNA expression vector, BglBrick vector, pMB1 *ori*, Spe^r^, sgRNA plasmid	Niu et al., 2018
pTargetF-RAG	pMB1 *ori*, Spe^r^, sgRNA-REG	This study
pSIM6	pSC101 replicon^ts^ P_L_-*gam*-*bet*-*exocI*857, Amp^r^	Sharan et al., 2009

### Plasmid Construction

The codon-optimized *tal* from *Rhodotorula glutinis*, *4cl* from *A. thaliana*, and *sts*/*romt* from *V. vinifera* were synthesized by GENEWIZ, Inc. (Suzhou, China). The *tal* fragment was amplified using the primer pair of tal-F/tal-R and digested with *Nhe*I/*Kpn*I. After digestion, the *tal* fragment was inserted into the *Nhe*I/*Kpn*I sites of the pZAC vector to form pZAC-tal. The *4cl* fragment was amplified using the primer pair of 4cl-F/4cl-R and digested with *Not*I/*Pst*I. The digested *4cl* fragment was then cloned into the *Not*I/*Pst*I sites of pZAC plasmid to obtain pZAC-4cl. For the construction of pZAC-tal-4cl, both pZAC-tal and pZAC-4cl were digested with *Kpn*I/*Bam*HI. The corresponding *tal*-containing fragment in pZAC-tal and *4cl*-containing fragment in pZAC-4cl were then ligated using T4 DNA ligase to build pZAC-tal-4cl. Similar to the construction of pZAC-tal, pZAC-4cl, and pZAC-tal-4cl, plasmids pZBK-sts, pZBK-romt, and pZBK-sts-romt were constructed accordingly due to the identical multiple clone sites (MCS) that pZBK shares with pZAC. Shortly, the *sts* fragment was amplified using the primer pair of sts-F/sts-R, followed by insertion into the *Nhe*I/*Kpn*I sites of pZBK to form pZBK-sts. Amplification of the *romt* fragment was performed using the primer pair of romt-F/romt-R, and then the *romt* fragment was cloned into the *Not*I/*Pst*I sites of pZBK to get pZBK-romt. Plasmid pZBK-sts-romt was obtained simply by inserting the *Nhe*I/*Kpn*I-digested *sts* fragment into the *Nhe*I/*Kpn*I sites of pZBK-romt. For the construction of pZBK-sts53-romt18, the evolved *sts* fragment was assembled onto the *Nhe*I/*Kpn*I sites of pZBK-romt18 *via* the digestion-ligation-dependent method, as described above.

### Generating Random Mutagenesis Library and Screening

MEGAWHOP (Miyazaki, 2003) was carried out for the construction of libraries. Firstly, the introduction of random mutagenesis into the corresponding gene or gene cluster was conducted *via* error-prone polymerase chain reaction (PCR). The PCR reaction mixture (50 μl) contained the following: 5 mM MgCl_2_, 0.3 mM MnCl_2_, 0.2 mM each of dATP, dGTP, dCTP, and dTTP, and 2.5 U of rTaq DNA polymerase. The PCR products then served as the megaprimers for performing MEGAWHOP (Miyazaki, 2003) to replace the wild-type gene or gene cluster with the derived mutants. After running MEGAWHOP, 20 U of *Dpn*I was added to the PCR reaction mixture and incubated overnight at 37°C to eliminate the surplus template. The *Dpn*I-treated PCR products were then transformed into *E. coli* DH5α and plated on LB agar containing the appropriate antibiotics to form the colony library. Then, sterilized normal saline was added to the agar plate and colonies were washed and pooled together so that a liquid library was achieved. Following the procedure of overnight culture and plasmid isolation, a plasmid-form library for subsequent transformation was obtained.

The mutant plasmid of the *tal*-*4cl* gene cluster was recovered from the library and transferred into the L-DOPA (L-3,4-dihydroxyphenylalanine) overproducer *E. coli* DOPA-30N (Wei et al., 2016). Then, the colonies were picked and inoculated into 48-deep-well microplates with each well containing 1 ml of the corresponding fermentation medium, followed by incubation at 37°C and 1,000 rpm on an MBR-420FL shaker (TAITEC Corporation, Saitama, Japan) for 24 or 48 h. Since the heterologous TAL-4CL pathway competes for the same precursor of L-tyrosine with the L-DOPA synthesis pathway, the light color of culture was selected for shake flask fermentation.

For screening the *sts* library, the *sts* mutant plasmids were co-transformed with the selected pZAC-tal-4cl variant p214C and a resveratrol biosensor pTtgR (Xiong et al., 2017) into *E. coli* TYR-30 for *de novo* resveratrol biosynthesis. After the abovementioned deep-well cultivation, cells in each well were washed twice with PBS solution (pH 7.0) and resuspended with an equal volume of PBS solution so that the cell suspension was obtained. Then, 200 μl of the cell suspension was transferred into a 96-well plate, followed by the determination of OD_600_ and the measurement of fluorescence using a SynergyNeo2 multimode reader (SynergyNeo2, BioTek, United States) with an excitation wavelength of 556 nm and an emission wavelength of 586 nm.

For screening the *romt* library, the *romt* mutant plasmids were co-transformed with the resveratrol biosensor pTtgR into *E. coli* BW25113. Colonies were inoculated into 48-deep-well microplates with each well containing LB medium supplemented with 0.5 mM of substrate resveratrol to check the resveratrol-to-pterostilbene conversion performance. In this experiment, a lower value of fluorescence/OD_600_ means more resveratrol has been transformed to pterostilbene due to stronger ROMT activity; thus, variants with lower fluorescence/OD_600_ value were selected and subjected to shake-flask verification.

### Knockout of Genomic Locus

Knockouts of gene or gene cluster in the *E. coli* genome were conducted by following the procedure described by Jiang et al. (2015). Plasmid pTargetF-RAG carrying the corresponding sgRNA with a specific N20 sequence targeting the aimed genomic locus was constructed. To eliminate a gene or gene cluster of interest, the approximate 500-bp DNA fragments directly upstream and downstream of this gene or gene cluster were amplified, respectively using the *E. coli* genome as the template. The amplified fragments were then fused by overlap PCR to construct an approximate 1,000-bp linear DNA fragment, which serves as the homologous arms for the genomic knockout. For conducting genomic deletion, plasmid pCas^∗^ was firstly transformed to the host strain for Cas 9 expression. The strain harboring pCas^∗^ was then inoculated into LB medium for the preparation of electrocompetent cells. Ten millimolars of arabinose was added to the culture medium to induce the expression of λ-Red recombinase. The prepared competent cells were then co-transformed with the corresponding pTargetF and linear fragment by electroporation. After 3 h of recovery, the electroporated strain was then harvested by centrifugation and plated on LB agar. Colonies with the desired genomic deletion were verified by colony PCR.

### Atmospheric and Room-Temperature Plasma Mutagenesis

A single colony carrying the corresponding biosensor (L-tyrosine biosensor pSen-tyr) was picked and grown in a falcon tube containing 5 ml of LB medium supplemented with appropriate antibiotics at 37°C and 200 rpm. One milliliter of the culture broth was then harvested by centrifugation when the optical density at 600 nm (OD_600_) approached about 0.8. The obtained cell pellet was washed twice with normal saline and finally resuspended with an equal volume of normal saline to obtain the cell suspension. Atmospheric and room-temperature plasma (ARTP) treatment of the cell suspensions was performed using an ARTP-generating system (ARTP-IIS, Tmaxtree Biotechnology Co., Ltd., Wuxi, China), as previously described (Niu et al., 2018, 2020b). The working parameters were defined as follows: (1) the input of radiofrequency power was set at 120 W; meanwhile, the flow of helium was set at 10 SLM. The distance between the exit of plasma torch nozzle and the slide was set at 2 mm; (2) the different ARTP-exposure durations were selected as 20, 30, 40, 50, 60, 70, 80, and 90 s. When the treatment was completed, mutant cells derived from ARTP were eluted from slides with normal saline. The eluted cells were plated on LB agar containing the appropriate antibiotics and grown overnight at 37°C to generate the colony library. Colonies on the agar plate were picked and inoculated into 48-deep-well microplates containing 1 ml of the MR/2 medium (Kim et al., 2018). The deep-well microplates were incubated at 37°C, 1,000 rpm, for 48 h on an MBR-420FL shaker (TAITEC, Nishikata, Japan). Cells were collected by centrifugation at 14,000 × *g* for 2 min, washed with PBS solution (pH 7.0) two times, and finally resuspended with an equal volume of PBS solution to obtain cell suspension. Two hundred microliters of the cell suspension was transferred into a 96-well plate, followed by the determination of OD_600_ and the measurement of fluorescence using a SynergyNeo2 multimode reader (SynergyNeo2, BioTek, Winooski, VT, United States) with an excitation wavelength of 535 nm and an emission wavelength of 610 nm.

### Error-*P*rone Polymerase Chain Reaction-*B*ased *W*hole *G*enome *S*huffling

Error-prone PCR-based whole genome shuffling (ep-WGS) was carried out as previously described (Ye et al., 2013; Niu et al., 2020b) with some modifications. Firstly, the error-prone amplification was conducted using the specific bacterial genome as the template. The PCR reaction mixture was set at a total volume of 50 μl consisting of 100 ng of genomic DNA, 0.2 mM of each dATP and dGTP, 1 mM of each dCTP and dTTP, 33.3 μM of 15-mer random primers, 5 mM of MgCl_2_, 0.3 mM of MnCl_2_, and 2 U of Taq DNA polymerase. The PCR reaction was performed as follows: 92°C for 1 min, 50 cycles at 37°C for 2 min, a programmed ramping of 0.1°C per s to 55°C, and 4 min extension at 55°C. The derived PCR products were then ethanol-precipitated and stored at −20°C for subsequent transformation.

*Escherichia coli* harboring pSIM6 was grown in 5 ml of LB medium supplemented with 100 μg/ml ampicillin at 30°C and 200 rpm until the OD_600_ reached 0.5–0.7. The cultures were heat-shocked in a shaking water bath at 42°C for 15 min to induce the expression of λ-Red recombinases. Then, the cells were made electrocompetent and transformed with the above ethanol-precipitated PCR products. After electroporation, the cells were plated on LB low salt medium and pooled together after overnight growth to obtain the library. The library was screened as described above.

### Shake-Flask Fermentation

Cells from a glycerol stock were streaked on LB plates and incubated for 24 h at 37°C. A single colony from the LB plate was inoculated into 5 ml LB medium in falcon tubes supplemented with the appropriate antibiotics, if necessary, which was cultured overnight at 37°C and 200 rpm. The seed cultures were transferred to 50 ml of the fermentation medium supplemented with the appropriate antibiotics, if necessary, with the initial OD_600_ of 0.1. For the *de novo* production of resveratrol and pterostilbene, a modified M9 medium with the supplementation of 15 g/l glucose and 10 ml/l of trace metal solution was used as the fermentation medium (1 mM of L-methionine was also supplied for pterostilbene production only to facilitate the methylation of resveratrol to form pterostilbene). The modified M 9 medium contains the following per liter: 12.8 g Na_2_HPO_4_⋅7H_2_O, 3 g KH_2_PO_4_, 0.5 g NaCl, 1 g NH_4_Cl, 10 g yeast extract, and 42 g MOPS. Trace metal solution contains the following per liter: 10 g FeSO_4_⋅7H_2_O, 1.35 g CaCl_2_, 2.2 g ZnSO_4_⋅7H_2_O, 0.58 g MnSO_4_⋅4H_2_O, 1 g CuSO_4_⋅5H_2_O, 0.1 g (NH4)_6_Mo_7_O_24_⋅4H_2_O, 0.2 g Na_2_B_4_O_7_⋅10H_2_O, and 10 ml of 35% (wt) HCl. The fermentation cultures were then incubated at 37°C and 200 rpm for 48 h. For the production of pterostilbene, 2 mM of *p*-coumaric acid was added to the medium. For the production of pterostilbene from resveratrol, 0.5 mM of resveratrol and 1 mM of L-methionine were added.

For L-tyrosine production, the baffled flask contained MR/2 medium reported by Kim et al. (2018) supplemented with 20 g/l of glucose and 5 ml/l of trace metal solution was used. The cultures were cultivated at 37°C and 200 rpm for 48 h. The MR/2 medium includes the following per liter: 6.67 g KH_2_PO_4_, 4 g (NH_4_)_2_HPO_4_, 0.8 g citric acid, 15 g (NH_4_)_2_SO_4_, 0.8 g MgSO_4_⋅7H_2_O, and 3 g yeast extract.

### Catalytic Efficiency Assays

*Escherichia coli* BW25113 harboring the responding plasmid was cultured in 5 ml LB medium in falcon tubes at 37°C and 200 rpm overnight. The overnight cultured cells were inoculated into 50 ml TB medium (tryptone 12 g/l, yeast extract 24.0 g/l, K_2_PO_4_ 9.4 g/l, K_3_PO_4_ 2.2 g/l) with the initial OD_600_ of 0.1. The cultures were incubated at 37°C and 200 rpm for 4 h and then at 20°C and 200 rpm for ∼20 h. The cells were centrifuged at 8,000 × *g* and 4°C for 15 min, and the resulting pellet was rinsed with ice-cold 0.85% NaCl solution twice. For *in vivo* activity assay, the cell pellets were resuspended in 10 ml phosphate buffer (0.1 M, pH 7.0) containing the responding substrate to form a cell suspension (OD_600_ = 40). The catalytic reaction was performed at 37°C and 200 rpm for 24 h. Samples were withdrawn every 2 h, and the reaction was stopped by ice-water bath cooling.

For *in vitro* activity assay, the cell pellets prepared as above were resuspended in 10 ml phosphate buffer (0.1 M, pH 7.0) (0.5 ml per gram cell pellet) and lysed using an Ultra-high Pressure Cell Disrupter (JN-3000Plus, Guangzhou Juneng Nano & Bio Technology Co., Ltd., China) at 1,500 bar. The lysate was clarified twice by centrifugation at 30,000 × *g* and 4°C for 30 min. The protein concentration was quantified by the Bradford assay, with bovine serum albumin as the standard using a SynergyNeo2 multimode reader (SynergyNeo2, BioTek, United States). The resultant supernatant was used as the crude extract. The crude extract was added in 10 ml PBS buffer (0.1 M, pH 7.0) containing the responding substrate. The protein concentration in this reaction mixture was kept as 10 mg/ml. The catalytic reaction was performed at 37°C and 200 rpm for 24 h. Samples were withdrawn every 2 h, and the reaction was stopped by ice-water bath cooling.

L-tyrosine (1 g/L), L-Tyr (1 g/L), and resveratrol (0.5 mM) were used as the substrate for assaying the activity of the TAL-4CL, STS, and ROMT, respectively. For assaying the activity of ROMT, 1 mM SAM was also added to the reaction mixture.

### Assay

Bacterial growth was measured using a UV-Visible spectrophotometer (GENESYS 180, Thermo Scientific, Waltham, MA, United States) and was indicated as the optical density at 600 nm (OD_600_).

All the metabolites were detected and quantified using a Shimadzu HPLC system (LC-20A, Shimadzu, Kyoto, Japan) equipped with an Inertsil ODS-SP column (5 μm, 4.6 × 150 mm, GL Sciences Inc., Tokyo, Japan). For the analysis of L-tyrosine, 100 μl of culture was diluted into 900 μl of 100 mM NaOH solution for full dissolution of produced L-tyrosine, followed by centrifugation at 12,000 × *g* for 2 min to obtain the supernatant. Trifluoroacetic acid (TFA) (0.2%) in water (Solution A) and absolute methanol (Solution B) were used as the mobile phases at a flow rate of 0.5 ml/min. A gradient procedure was used: 14–45% B (0–20 min) and 14% (20–30 min). The column temperature was maintained at 30°C, and a photodiode array detector (SPDM20A) operating at 280 nm was used. For analysis of pterostilbene and resveratrol, 500 μl of culture was extracted with an equal volume of ethyl acetate. The ethyl acetate was dried, and the sample was resuspended in 500 μl of methanol. The concentrations were measured by the HPLC method reported by Wang et al. (2015). Formic acid (0.1%) in water (Solution A) and absolute acetonitrile (Solution B) were used as elution phases with a flow rate of 0.8 ml/min. The gradient elution program is as follows: 10% B (0–2 min), 10–70% B (2–20 min), 30% B (20–21 min), 10% B (21–23 min), and 10% B (23–28 min). The column temperature was maintained at 40°C. They were detected using a photodiode array detector (SPDM20A) operating at 300 nm. Quantification of the abovementioned metabolites was conducted by consulting the corresponding standard curves derived from HPLC analysis of the serially diluted standard stock solutions.

### Statistical Analysis

All the fermentative experiments in this study were conducted in triplicate, and the data were presented as the means ± standard deviations. One-way analysis of variance followed by Tukey’s test was employed to check significant differences using the OriginPro (version 8.0) package. Statistical significance was defined as *p* < 0.05.

## Results and Discussion

### Directed Evolution of Heterologous Pathway Enzymes

Santos et al. (2011) introduced the *tal* from *R. glutinis* for converting the endogenous L-tyrosine to *p*-coumaric acid. Lim et al. (2011) compared *4cl* and *sts* from different organisms and found that the *4cl* from *A. thaliana* and *sts* from *V. vinifera* yielded the highest resveratrol titer to 2.3 g/l. Wang et al. (2015) reported that the highest conversion efficiency of resveratrol to pterostilbene was obtained using the ROMT from *V. vinifera*. Thus, *R. glutinis tal* (*Rgtal*), *A. thaliana 4cl* (*At4cl*), *V. vinifera sts* (*Vvsts*), and *V. vinifera romt* (*Vvromt*) were selected to establish the biosynthetic pathway of pterostilbene (Figure 1). Zhou et al. (2016) reported that the *Rg*TAL (S9N/A11T/E518V) mutant exhibited higher catalytic activity on L-Tyr than the wild-type enzyme. Thus, the *Rg*TAL mutant was used in this study. To achieve *de novo* production of pterostilbene in *E. coli*, four heterologous enzyme genes were cloned into the pZAC and pZBK vectors to obtain the expression plasmids, pZAC-tal-4cl and pZBK-sts-romt, respectively. Then, the two plasmids were transformed into an L-Tyr-producing strain *E. coli* TYR-30 which led to production of 2.39 ± 0.31 mg/l (Table 2) pterostilbene.

**TABLE 2:** *De novo* production of pterostilbene in *E. coli*.

Plasmids	Host	Pterostilbene (mg/L)	OD_600_
pZAC-tal-4cl, pZBK-sts-romt	*E. coli* TYR-30	2.39 ± 0.31	10.10 ± 0.22
p214C, pZBK-sts-romt	*E. coli* TYR-30	17.48 ± 1.09	10.70 ± 0.23
p214C, pZBK-sts53-romt18	*E. coli* TYR-30	32.75 ± 2.12	10.41 ± 0.09
p214C, pZBK-sts53-romt18	*E. coli* TYR-14B1	80.04 ± 5.58	10.13 ± 0.01

*The data are presented as means of three replicates with its corresponding standard deviations.*

**FIGURE 2:**
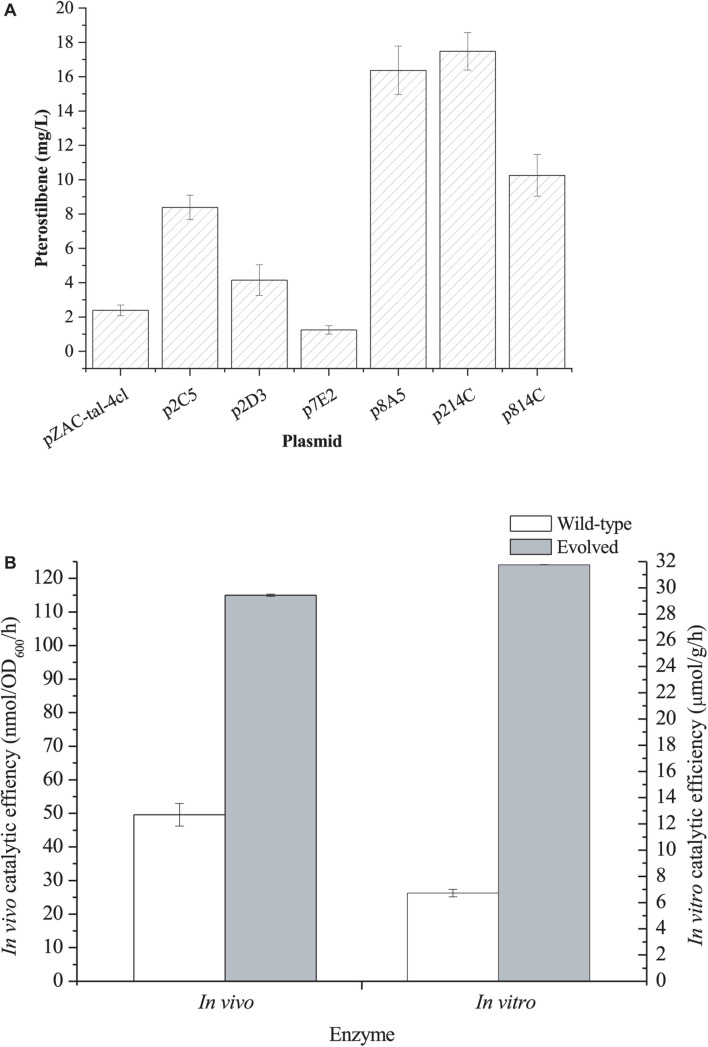
**(A)** *De novo* pterostilbene production by *E. coli* TYR-30 harboring the mutant plasmids of pZAC-tal-4cl and pZBK-sts-romt. **(B)** *In vivo* and *In vitro* catalytic efficiency of the evolved pathway enzymes of TAL-4CL and its wild-type enzymes. *In vivo* activity was defined as the L-tyrosine consuming rate of the whole resting cells and *In vitro* activity was defined as the L-tyrosine consuming rate of the crude extract. The data represent means of three replicates and error bars represent standard deviations.

In general, the heterologous pathway enzyme shows low activity in the heterologous host strain. To increase the catalytic efficiency, we first directly co-evolved the *Rgtal-At4cl* gene cluster by error PCR. The mutant plasmids were transformed into the L-DOPA overproducer *E. coli* DOPA-30N. Since the synthesized L-DOPA can be easily oxidized to dopachrome and then non-enzymatically polymerized to form melanin (Shen et al., 2019), it results in the black color of the culture broth. Because the formation of pterostilbene utilizes the same precursors (L-Tyr) as the L-DOPA biosynthesis, the pterostilbene biosynthesis competes with L-DOPA. The higher the catalytic efficiency of the gene cluster, the lower the production of L-DOPA, hence the culture broth becomes lighter. A total of 480 colonies were selected for the deep-well screening. Of them, six strains with lighter colors of the culture broth were selected for further shake flask fermentation. The six mutant plasmids were recovered and individually transformed an L-Tyr-producing strain *E. coli* TYR-30 harboring pZBK-sts-romt for the shake flask fermentation of the pterostilbene biosynthesis. As shown in Figure 2A, the strain harboring the mutant plasmid p214C produced the highest pterostilbene production of 17.48 ± 1.09 mg/l. The sequencing results of the mutant plasmid p214C are shown in Supplementary Table 2. Three base substitutions (A486T/A669G/A894G), which result in no amino acid mutant, were found in the *tal*. Three base substitutions (G45A/C169A/T1379A), which result in two amino acid mutants (L57I/L460H), were observed in the *4cl*. Xiong et al. (2017) applied a biosensor of resveratrol and screened a 4CL mutant (I250L/N404K/I461V), in which the catalytic activity on *p*-coumaric acid was 1.7-fold higher than the wild-type enzyme. To evaluate the effect of the evolved enzymes on the production of pterostilbene, we compared the *in vivo* and *in vitro* activity of the pathway enzymes. As shown in Figure 2B, the directed evolution resulted in an increase in the *in vitro* and *in vivo* activities of the pathway enzyme to 31.76 ± 0.01 μmol/h/g protein from 6.72 ± 0.28 μmol/h/g protein and 115.00 ± 0.32 nmol/OD/h from 49.61 ± 3.35 nmol/OD/h, respectively. This result indicates that the evolved pathway was more efficient at converting L-Tyr than the wild-type pathway both *in vitro* and *in vivo*. Homologous modeling revealed that mutation L57I was far away from the active site (>20 Å) and thus may not be related to the activity enhancement (Supplementary Figure 1). Mutation L460H was located at the entrance of the active site (Supplementary Figure 1). After mutation, the residue comes closer to the substrate, which may form a hydrogen bond with the substrate and thus boost catalysis.

**FIGURE 3:**
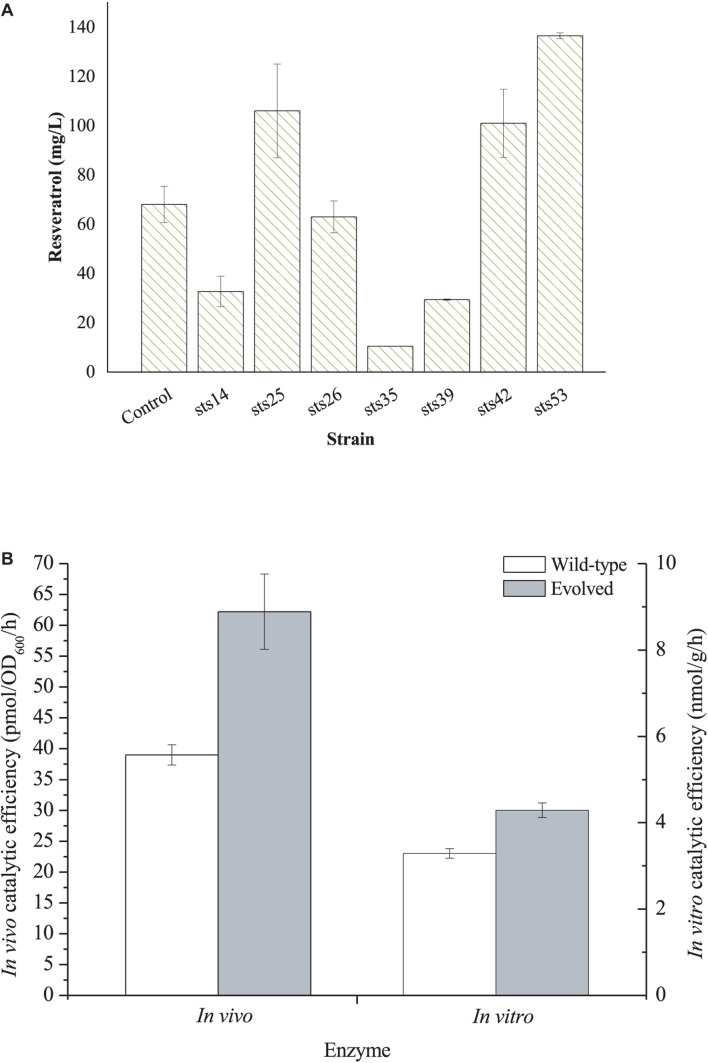
**(A)** *De novo* resveratrol production by *E. coli* TYR-30 harboring the mutant plasmids of pZBK-sts and p214C. *E. coli* TYR-30 harboring pZBK-sts and p214C was set as the control. **(B)** *In vivo* and *In vitro* catalytic efficiency of the evolved STS and its wild-type enzyme. *In vivo* activity was defined as the resveratrol forming rate of the whole resting cells and *In vitro* activity was defined as the resveratrol forming rate of the crude extract. The data represent means of three replicates and error bars represent standard deviations.

For directly evolving STS, the mutant *sts* fragments obtained through error-prone PCR were cloned into the pZBK vector by MEGAWHOP (Miyazaki, 2003). Then, the plasmid library of *sts* variants was transformed into an L-Tyr-overproducing strain *E. coli* TYR-30 harboring p214C and the resveratrol biosensor plasmid pTtgR to check for *de novo* resveratrol biosynthesis. The fluorescence of the biosensor is positively corrected with resveratrol production, indicating that we can measure the resveratrol production by monitoring the fluorescence strength. A total of 1,102 colonies were selected for the deep-well analysis (Supplementary Figure 2). Seven strains with higher fluorescence strength were selected for further shake flask fermentation. The seven mutant plasmids were recovered and individually transformed into an L-Tyr-producing strain *E. coli* TYR-30 harboring p214C for the shake flask fermentation of the resveratrol biosynthesis. As shown in Figure 3A, strain sts53 produced the highest level of resveratrol, which reached 136.59 ± 1.18 mg/l. The resveratrol titer was about two-fold that (68.05 ± 7.37 mg/l) harboring the wild-type STS. The mutant plasmid was recovered from the strain sts53 and was named pZBK-sts53. The sequencing results of the mutant plasmid pZBK-sts53 are shown in Supplementary Table 2. Two base substitutions (C149T/T509C), which result in two amino acid mutants (T50I/V170A) were observed in the *sts*. To evaluate the effect of the evolved enzyme on the production of pterostilbene, we determined the *in vivo* and *in vitro* activities of the mutant and compared them with the wild-type enzyme. As shown in Figure 3B, the directed evolution resulted in an increase in the *in vitro* and *in vivo* activities of the pathway enzyme to 4.29 ± 0.17 nmol/h/g protein from 3.29 ± 0.11 nmol/h/g protein and 62.22 ± 6.10 pmol/OD_600_/h from 39.00 ± 1.64 pmol/OD_600_/h, respectively. This result indicates that the evolved enzyme was more efficient at the production of resveratrol than the wild-type enzyme both *in vitro* and *in vivo*. Homologous modeling revealed that mutation T50I was far away from the active site (>20 Å) and thus may not be related to the activity enhancement (Supplementary Figure 3). Mutation V170A was located at the bottom of the active site (Supplementary Figure 3). After mutation, the residue becomes smaller, which may expand the active site to better accommodate the substrate, thereby increasing the activity.

**FIGURE 4:**
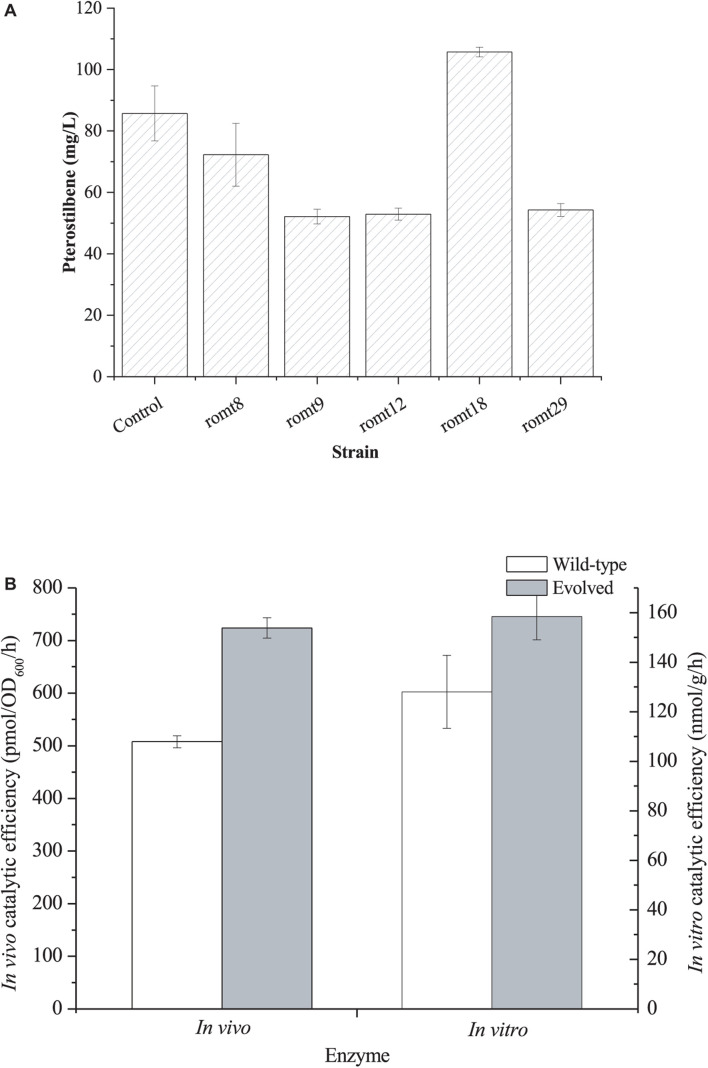
**(A)** Production of pterostilbene from resveratrol by *E. coli* BW25113 harboring the mutant plasmids of pZBK-romt. *E. coli* BW25113 harboring pZBK-romt was set as the control. **(B)** *In vivo* and *In vitro* catalytic efficiency of the evolved ROMT and its wild-type enzyme. *In vivo* activity was defined as the pterostilbene forming rate of the whole resting cells and *In vitro* activity was defined as t the pterostilbene forming rate of the crude extract. The data represent means of three replicates and error bars represent standard deviations.

Finally, we directly evolved VvROMT *via* error-PCR. The evolved library was transformed into *E. coli* BW25113 harboring the resveratrol biosensor pTtgR. A total of 920 colonies were selected for deep-well analysis (Supplementary Figure 4). With the aid of the resveratrol biosensor pTtgR, five strains with lower fluorescence strength were selected for further shake flask fermentation of pterostilbene biosynthesis. As shown in Figure 4A, strain romt18 produced the highest level of pterostilbene from resveratrol. The mutant plasmid was recovered from the strain romt18 and was named pZBK-romt18. The sequencing results of the mutant plasmid pZBK-romt18 are shown in Supplementary Table 2. Four base substitutions (T18G/T85C/T294C/T477C), which result in one amino acid mutant (S29P), were observed in the *romt*. We also assayed the *in vivo* and *in vitro* activities of the mutant and compared them with the wild-type enzyme. As shown in Figure 4B, the directed evolution resulted in an increase in the *in vitro* and *in vivo* activities of the pathway enzyme to 158.43 ± 9.44 nmol/h/g protein from 128.01 ± 14.71 nmol/h/g protein and 723.87 ± 19.47 pmol/OD_600_/h from 507.80 ± 11.50 pmol/OD_600_/h, respectively. This result indicates that the evolved enzyme was more efficient at the production of pterostilbene than the wild-type enzyme both *in vitro* and *in vivo*. Homologous modeling revealed that the enzyme is a homodimer and the mutation S29P was located on the interface (Supplementary Figure 5A). After mutation, four hydrogen bonds between S29 with H22 (3.4 Å), I23 (2.8 Å), N25 (3.2 Å), and F26 (3.1 Å) of the other chain were lost (Supplementary Figure 5B), which may make the N-terminal domain of the enzyme more flexible and thus facilitate the substrate binding.

The mutant gene *romt18* was cut from pZBK-romt18 and cloned into pZBK-sts53 to obtain pZBK-sts53-romt18. The plasmid pZBK-sts53-romt18 was transformed into an L-Tyr-overproducing strain *E. coli* TYR-30 harboring p214C for the shake flask fermentation of the pterostilbene biosynthesis. As shown in Table 2, the resulting strain produced 32.75 ± 2.12 mg/l, which is 13.7-fold the strain harboring the wild-type pathway.

In this study, four evolved enzymes (Supplementary Table 2) that outperformed the wild-type enzymes were obtained after the directed evolution. The directed evolution of the pathway enzymes increased the pterostilbene production by 13.7-fold (Table 2).

### Host Strain Engineering Increasing the Availability of L-Tyrosine

**FIGURE 5:**
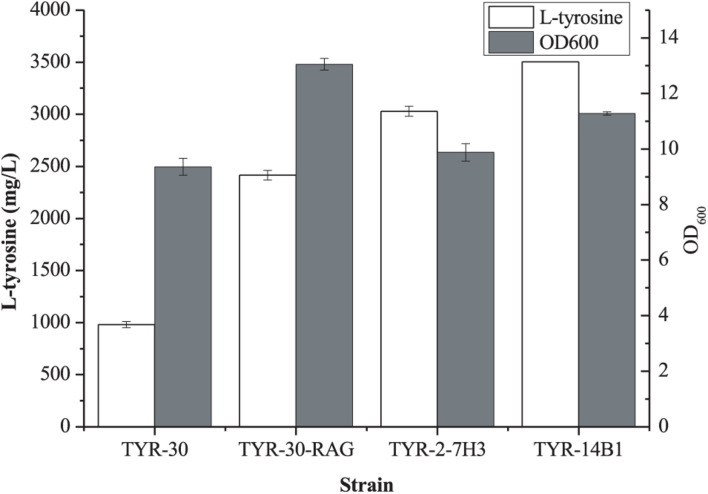
Production of L-tyrosine of the selected *E. coli* strains. The data represent means of three replicates and error bars represent standard deviations.

Previous studies demonstrated that increasing the availability of L-Tyr improved the production of aromatic compounds (Li et al., 2016; Heo et al., 2017; Niu et al., 2020a; Shen et al., 2020). Aguilar et al. (2012, 2015) reported that deletion of the 2-acyl-glycerophosphoethanolamine cycle allows fast growth in glucose and improves the production of aromatic compounds in *E. coli* strain lacking the phosphoenolpyruvate: carbohydrate phosphotransferase system (PTS). The 2-acyl-glycerophosphoethanolamine cycle comprises 12 contiguous genes (*rppH*, *ygdT*, *mutH*, *ygdQ*, *ygdR*, *tas*, *lplT*, *aas*, *omrB*, and part of *ptsP and galR*, 10,328 bp). This growth advantage may be the increase of mRNA levels of glycolytic genes due to RppH pyrophosphohydrolase activity deficiency and the increased glucose uptake through GalP because of the lack of GalR (Aguilar et al., 2012, 2015). The starting strain *E. coli* TYR-30 in this study is a derivative of *E. coli* DOPA-30N which is lacking PTS (Wei et al., 2016). Thus, we first deleted the 2-acyl-glycerophosphoethanolamine cycle (10328 bp) in *E. coli* TYR-30 to obtain *E. coli* TYR-30-RAG. As shown in Figure 5, the deletion of the 2-acyl-glycerophosphoethanolamine cycle enhanced the L-Tyr titer to 2415.68 ± 47.72 mg/l from 981.24 ± 29.72 mg/l.

**FIGURE 6:**
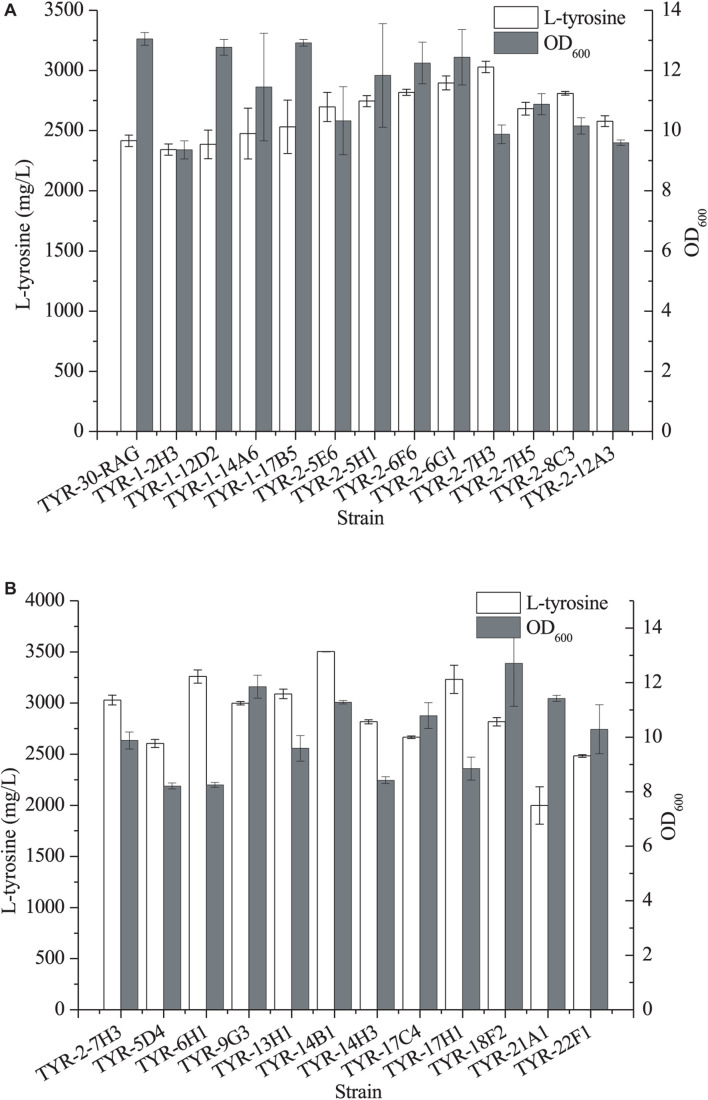
Production of L-tyrosine of the selected *E. coli* strains after ARTP mutagenesis **(A)** and error-prone whole genome shuffling **(B)**. The data represent means of three replicates and error bars represent standard deviations.

In our previous study (Niu et al., 2020b), we developed a strain improvement strategy of biosensor-guided genome shuffling and applied this strategy to improve the production of shikimic acid in *E. coli*. Thus, we applied this strategy to improve the availability of L-Tyr using the L-Tyr biosensor pSen-tyr (Supplementary Figure 6). ARTP is a rapid, effective mutagenesis tool (Zhang et al., 2014; Niu et al., 2020b). Thus, we first applied ARTP mutagenesis to generate a microbial mutant library. After *E. coli* TYR-30-RAG cells were treated with ARTP, the L-Tyr biosensor pSen-tyr was transformed into the mutant strains. A total of 1,007 colonies were selected for deep-well analysis (Supplementary Figure 7). Twelve strains with higher fluorescence strength were screened out for further shake flask fermentation. As shown in Figure 6A, the *E. coli* TYR-2-7H3 strain produced the highest titer of L-tyrosine, which achieved 3029.18 ± 47.72 mg/l.

Error-prone PCR-based whole genome shuffling is a powerful technology for strain improvement that does not require knowledge of the genetic background of the microorganism. Moreover, it requires only one strain as the starting strain (Luhe et al., 2011). A total of 1,243 colonies were selected for deep-well analysis (Supplementary Figure 8). Eleven strains with higher fluorescence strength were screened out for further shake flask fermentation. As shown in Figure 6B, four strains exhibited the better production performance of L-tyrosine than the parental strain. Among them, *E. coli* TYR-14B1 strain produced the largest amount of L-tyrosine, which reached 3502.76 ± 0.28 mg/l.

We then examined the genetic stability of the obtained L-tyrosine-overproducing variants through constant subculture. The growth and L-Tyr titers of the three strains did not show significantly different after 25 rounds of subculturing (Supplementary Table 3). It indicates that these strains are genetically stable in the production of L-Tyr and growth.

### *De novo* Production of Pterostilbene From Glucose

Finally, we synthesized pterostilbene from glucose using *E. coli*. The evolved pathway was introduced into the shuffled strain *E. coli* TYR-14B1. In this system, *E. coli* TYR-14B1 produces L-tyrosine and then is converted into pterostilbene by the evolved pathway enzymes. As shown in Table 2, *E. coli* TYR-14B1 harboring the evolved pathway produced 80.04 ± 5.58 mg/l pterostilbene, which is about 2.3-fold the highest titer (34.93 mg/l) reported in literatures (Li et al., 2016). It indicates that the host strain engineering using the strategy of the biosensor-guided shuffling increased the production of pterostilbene by 1.4-fold compared to that obtained by *E. coli* YTR-30 harboring the evolved pathway. It was also found that the resulting strain produced pterostilbene as the sole methylated resveratrol and pinostilbene was not detected (Supplementary Figure 9).

## Conclusion

In this study, we first directly evolved the heterologous biosynthetic pathway enzyme of pterostilbene. Four mutant enzymes that outperformed the wild-type enzymes were obtained. The *in vitro* and *in vivo* activities of the evolved TAL-4CL was increased by 3.7-fold and 1.3-fold compared with the wild-type enzyme, respectively. The evolution resulted in an increase in *in vitro* and *in vivo* activities of STS by 30 and 60%, respectively. The *in vitro* and *in vivo* activities of the ROMT were increased by 24 and 43% after the directed evolution, respectively. The strain harboring these evolved pathway enzymes produced 13.7-fold pterostilbene higher that harboring the wild-type pathway.

We then applied the strategy of biosensor-guided genome shuffling to improve the production of L-Tyr in *E. coli*. The titer of L-Tyr was increased by 45%. Using the shuffled strain as the host of the evolved pathway enzymes further improved the production of pterostilbene by 1.4-fold, which achieved 80.04 ± 5.58 mg/l.

## Data Availability Statement

The original contributions presented in the study are included in the article/Supplementary Material, further inquiries can be directed to the corresponding author/s.

## Author Contributions

Z-BY wrote the manuscript and conducted most of the experiments involved in this work except for the following mentioned. J-LL constructed the plasmid pZAC-tal-4cl and performed the co-evolution of TAL-4CL. F-XN carried out the genomic deletion of RAG. Y-PS constructed the L-tyrosine biosensor pSen-tyr. J-ZL designed the study. All authors read and approved the final manuscript.

## Conflict of Interest

The authors declare that the research was conducted in the absence of any commercial or financial relationships that could be construed as a potential conflict of interest.

## Publisher’s Note

All claims expressed in this article are solely those of the authors and do not necessarily represent those of their affiliated organizations, or those of the publisher, the editors and the reviewers. Any product that may be evaluated in this article, or claim that may be made by its manufacturer, is not guaranteed or endorsed by the publisher.
