# Supplementary material for: Enhanced Production of Pterostilbene in Escherichia coli Through Directed Evolution and Host Strain Engineering
Source: Front Microbiol. 2021 Oct 7;12:710405. doi: 10.3389/fmicb.2021.710405 (PMC8530161; doi:10.3389/fmicb.2021.710405)
Supplement: Supplementary file 1 [file Data_Sheet_1.docx]

**Enhanced the production of pterostilbene in *Escherichia coli* through directed evolution and host strain engineering**

***Zhi-Bo Yan^1^, Jing-Long Liang^1,2^, Fu-Xing Niu^1^, Yu-Ping Shen^1^and Jian-Zhong Liu^1^****

*1, Institute of Synthetic Biology, Biomedical Center,* *Guangdong Province Key Laboratory of* *Improved Variety Reproduction in Aquatic Economic Animals, School of Life Sciences,* *Sun Yat-sen University, Guangzhou 510275, China*

*2, College of Light Industry and Food Science, Zhongkai University of Agriculture and Engineering, Guangzhou, China, 510225*

Email:

Zhi-Bo Yan, yanzhb3@mail2.sysu.edu.cn

Jing-Long Liang: liangjlong3@mail.sysu.edu.cn

Fu-Xing Niu: [niufx3@mail.sysu.edu.cn](mailto:niufx3@mail.sysu.edu.cn)

Yu-Ping Shen: shenyup@mail2.sysu.edu.cn

Jian-Zhong Liu: lssljz@mail.sysu.edu.cn

*Corresponding author: Institute of Synthetic Biology, School of Life Science, Sun Yat-Sen

University, Guangzhou 510275, P.R. China. Phone: +86-20-84110115. Fax: +86-20-84036461. *E-mail address*: lssljz@mail.sysu.edu.cn (J. Z. Liu)

# Supplementary Table 1 Primers used in this study

| **Name** | **Sequence (from 5’ to 3’)** | **Purpose** |
| --- | --- | --- |
| tal-F | CTAGCTAGCTTTCGGAATTAAGGAGGTAATAAATATGGCTCCTCGTCCTAC (NheI) | For normal amplification of *tal* |
| tal-R | CGGGGTACCTTATGCCAACATTTTTAGTAGAACA (KpnI) |  |
| 4cl-F | ATTTGCGGCCGCTTTCGGAATTAAGGAGGTAATAAATATGGCTCCACAAGAACAAGC (NotI) | For normal amplification of *4cl* |
| 4cl-R | CCAATGCATTGGTTCTGCAGTTACAAACCATTTGCTAG (PstI) |  |
| sts-F | CTAGCTAGCTTTCGGAATTAAGGAGGTAATAAATATGGCTTCAGTAGAAG (NheI) | For normal amplification of *sts* |
| sts-R | CGGGGTACCTTAATTTGTAACCATAGGAATAC (KpnI) |  |
| romt-F | ATTTGCGGCCGCTTTCGGAATTAAGGAGGTAATAAATATGGATTTGGCAAATGG (NotI) | For normal amplification of *romt* |
| romt-R | CCAATGCATTGGTTCTGCAGTTAAGGATAAACTTC (PstI) |  |
| ep_tal-4cl-F | ATGGCTCCTCGTCCTACTTC | For error-prone amplification of *tal*-*4cl* |
| ep_tal-4cl-R | TTACAAACCATTTGCTAGTTTTG |  |
| ep_sts/romt-F | GGTATATGTCATTTTTCTAG | For error-prone amplification of *sts* and *romt* |
| ep_sts-R | CCCTGCAGAGTACTAGC |  |
| ep_romt-R | GCACTAGTAGATCCGATATC |  |
| N20-rppH-F | GGACTAGTTCCTGGCAATTTCCGCAAGGGTTTTAGAGCTAGAAATAG | For constructing pTargetFs |
| N20-hpaC-F | GGACTAGTCCTGCGCTTTCGTGACGCGAGTTTTAGAGCTAGAAATAG |  |
| pTargetF-R | GGACTAGTATTATACCTAGGACTGAGCTAGCTGTCAAG |  |
| US-hpaBC-F | TGGTGAGCAATTCGTTCTCGAAAATTTTCTCCCTTTACGGCGAGCGCGTCGGCGG | For constructing the upstream homologous arm of *hpaBC* cluster |
| US-hpaBC-R | GCCGCCCCGGGGCTGGGCCCCGAACCACCGCCGCTTCCAC |  |
| DS-hpaBC-F | GTGGAAGCGGCGGTGGTTCGGGGCCCAGCCCCGGGGCGGC | For constructing the downstream homologous arm of *hpaBC* cluster |
| DS-hpaBC-R | CTGAACAAATCCAGATGGAGTTCTGAGGTCATTACTGGATCTATCAACAGGAGTC |  |
| US-RAG-F | CCGCTCGAGCGATACCCAAGCCCAATGAG | For constructing the upstream homologous arm of *RAG* cluster |
| US-RAG-R | TTGTGGTAACCGTTGCCAATCAGGTTGGCGTGGGATTCGCATTA |  |
| DS-RAG-F | TAATGCGAATCCCACGCCAACCTGATTGGCAACGGTTACCACAA | For constructing the downstream homologous arm of *RAG* cluster |
| DS-RAG-R | TATCCTAGGATTATCATTGAGAACGCCCAT |  |

# Supplementary Table 2 Summary of the introduced base mutations and the resultant amino acid change(s) in the evolved heterologous genes

| **Evolved genes** | **Base mutations** | **Amino acid change(s)** |
| --- | --- | --- |
| *tal*^214C^ | A486T, A669G, A894G | NO |
| *4cl*^214C^ | G45A, C169A, T1379A | L57I, L460H |
| *sts53* | C149T, T509C | T50I, V170A |
| *romt18* | T18G, T85C, T294C, T477C | S29P |

# Supplementary Table 3 Genetic stability of the shuffled strains

| **Strains/number subculture** | **L-tyrosine (mg/L)** | **OD_600_** |
| --- | --- | --- |
| ***E. coli* TYR-6H1** | | |
| 0 | 3259.41 ± 63.65 | 7.88 ± 1.16 |
| 25G | 3177.28 ± 20.22 | 8.03 ± 0.32 |
| ***E. coli* TYR-14B1** | | |
| 0 | 3480.87 ± 39.39 | 10.89 ± 0.09 |
| 5 | 3546.37 ± 62.01 | 10.20 ± 0.39 |
| 10 | 3478.73 ± 281.23 | 10.23 ± 0.57 |
| 15 | 3484.03 ± 18.45 | 10.44 ± 0.09 |
| 20 | 3702.20 ± 199.16 | 10.67 ± 0.20 |
| 25 | 3556.40 ± 102.53 | 10.22 ± 0.14 |
| ***E. coli* TYR-17H1** | | |
| 0 | 3231.96 ± 138.62 | 8.85 ± 0.42 |
| 25 | 3298.83 ± 12.24 | 8.51 ± 0.11 |


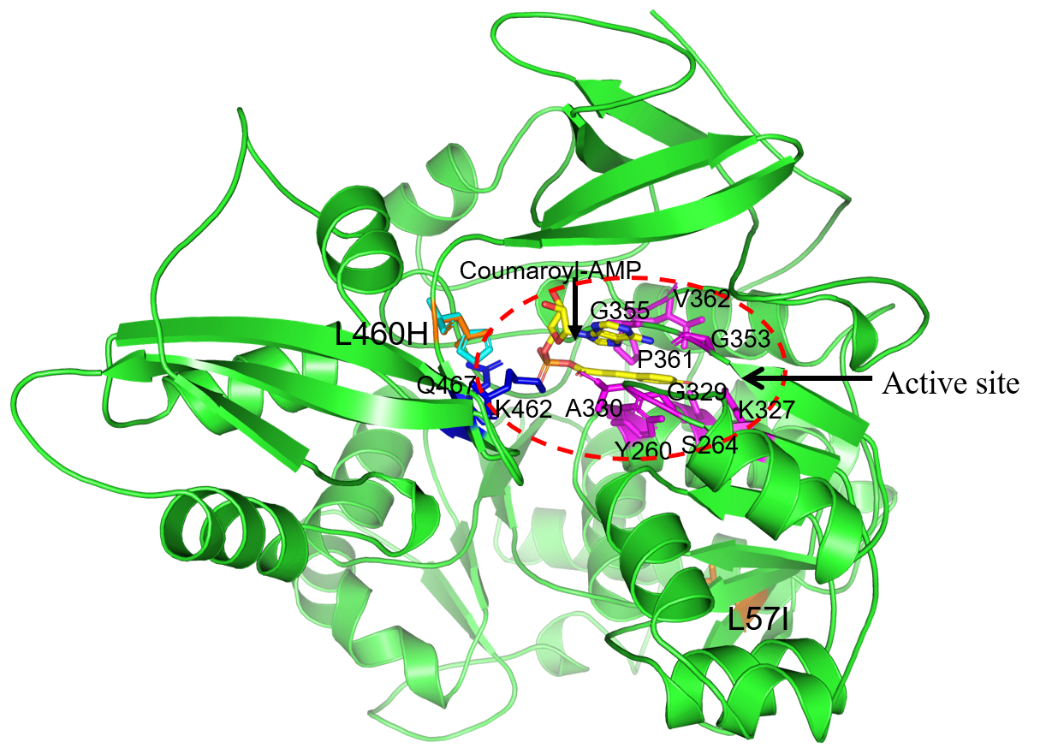


**Supplementary Figure 1** The locations of the mutated sites in the crystal structure of At4CL. Homologous modeling was performed by Swiss-Model (<https://swissmodel.expasy.org>) using the 4-coumarate:CoA ligase (4CL) isoform 2 (Nt4CL2) from *Nicotiana tabacum* as template (PDB: 5BSR) (Li and Nair, 2015). Their sequence identity was 72.57%. Mutations L57I was far away from the active site (>20 Å) and thus may not be related to the activity enhancement (figure ). Mutation L460H was located at the entrance of the active site (figure ). After mutation, the residue comes closer to the substrate, which may form hydrogen bond with the substrate, and thus boost catalysis. The key residues for substrate binding (Y260, S264, K327, G329, A330, G353, G355, P361 and V362) and catalysis (K462, Q467) reported by Hu et al. (2010), were shown as purple and blue sticks. L460 was shown as origine sticks, and H460 was shown as cyans sticks. The substrate coumaroyl-AMP was extracted from the structure of Nt4CL2 (PDB: 5BSR). The predicted active site was marked by a red circle.

**Supplementary Figure 2** The fluorescent strength of the mutant library of STS


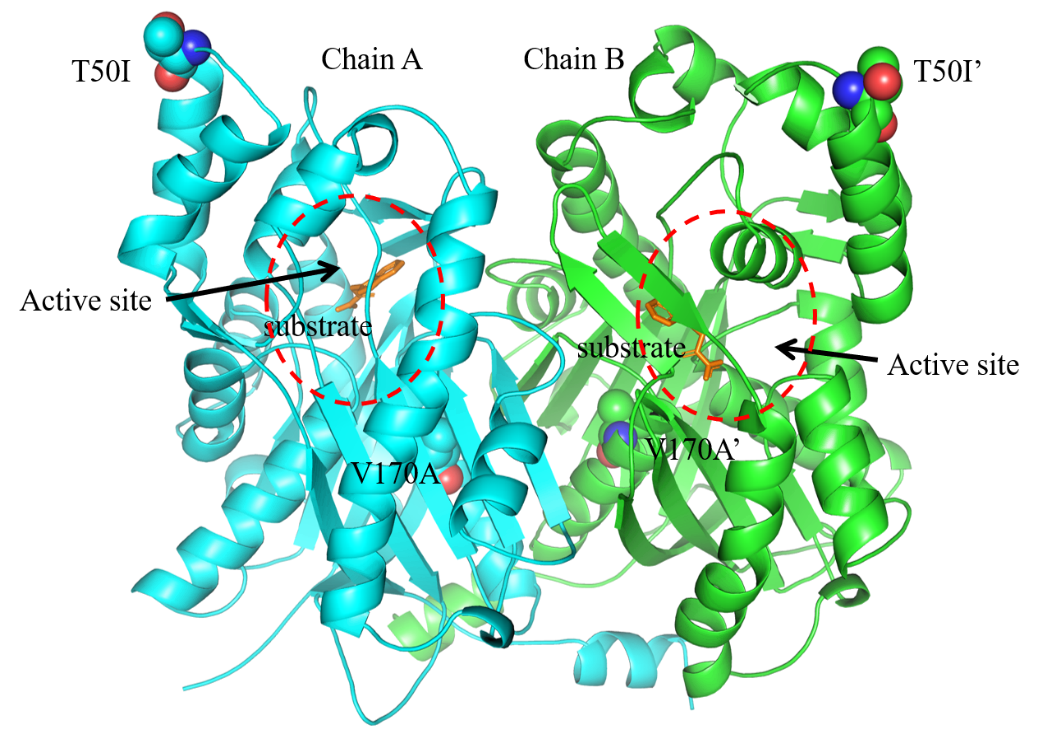


**Supplementary Figure 3** The locations of the mutated sites in the crystal structure of VvSTS. Homologous modeling was performed by Swiss-Model (<https://swissmodel.expasy.org>) using the stilbene synthase from *V. vinifera* (VvSTS) as template (PDB: 3TSY) (Wang et al., 2011). Their sequence identity was 99.23%. The enzyme is a homodimer. Mutations T50I was far away from the active site (>20 Å) and thus may not be related to the activity enhancement (figure ). Mutation V170A was located at the bottom of the active site (figure ). After mutation, the residue becomes smaller, which may expand the active site to better accommodate the substrate, thereby increasing the activity. The residues mutated were shown as spheres. The substrate 3-(1h-indol-3-yl)-2-oxopropanoic acid was extracted from the structure of a pine STS (PDB: 1XES). The predicted active site was marked by a red circle.

**Supplementary Figure 4** The fluorescent strength of the mutant library of ROMT


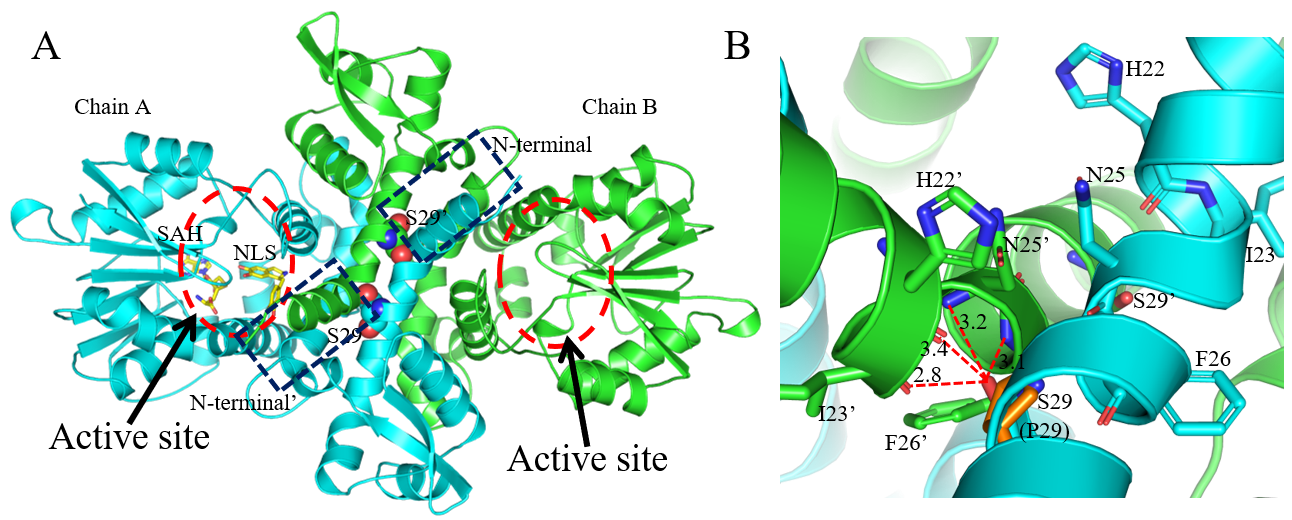


**Supplementary Figure 5** The locations of the mutated sites in the crystal structure of VvROMT. Homologous modeling was performed by Swiss-Model (<https://swissmodel.expasy.org>) using the (S)-norcoclaurine-6-O-methyltransferase (6OMT) from *Thalictrum flavum* as template (PDB: 5ICC) (Robin et al., 2016). Their sequence identity was 45.75%. The enzyme is a homodimer and the mutation S29P was located on the interface (A). After mutation, four hydrogen bonds between S29 with H22 (3.4 Å), I23 (2.8 Å), N25(3.2 Å) and F26 (3.1 Å) of the other chain were lost (B), which may make the N-terminal domain of the enzyme more flexible and thus facilitate the substeate binding. The residues mutated were shown as spheres in (A) and the residues formed hydrogen bonds with S29(S29’) were shown as sticks (B). The substrates S-adenosyl-L-homocysteine (SAH) and (R,S)-norlaudanosoline (NSL) were extrated from the structure of 6OMT (PDB: 5ICC). The predicted active sites were marked by red cirles. The N-terminals of the enzyme were marked by dark blue boxes. Possible hydrogen bonds were shown by red dashed lines, with distances shown in black (in angstroms).


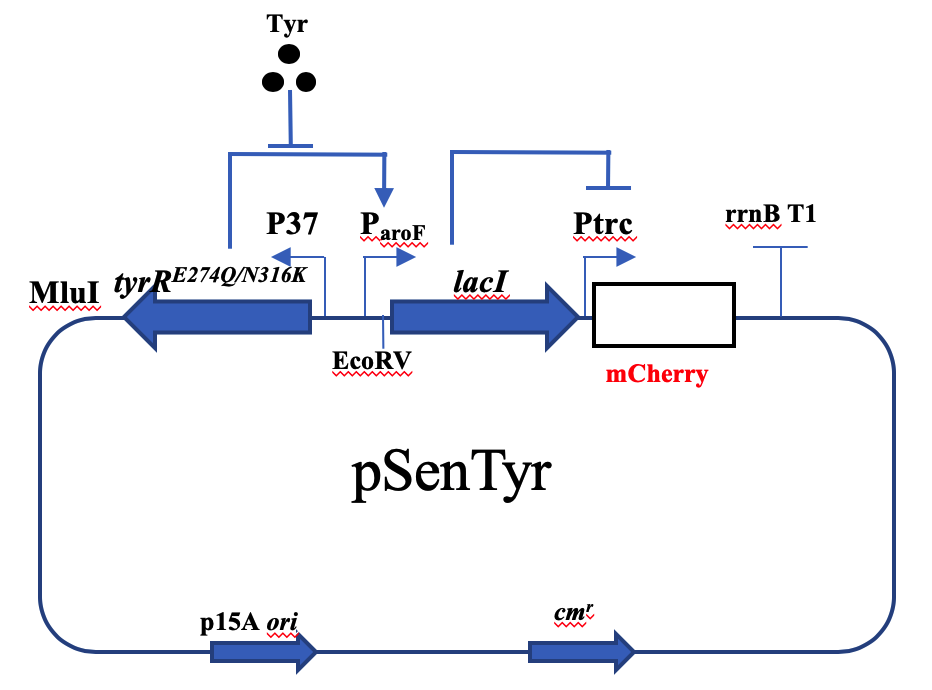


**Supplementary Figure 6** Plasmid map of the L-Tyr biosensor pSen-tyr constructed as previously described (Chou and Keasling, 2013). In the presence of L-tyrosine, L-tyrosine binds TyrR^E274Q/N316K^ to repress the expression of *lacI*, then the P_trc_ promoter drives the expression of mCherry. The fluorescence of the biosensor is positively corrected with L-tyrosine concentration

**Supplementary Figure 7** The fluorescent strength of the ARTP mutant strains

**Supplementary Figure 8** The fluorescent strength of the ep-WGS strains

**Supplementary Figure 9** HPLC profile of pterostilbene. Standard (A), the broth of *E. coli* BW25113 (pZBK-romt18) (B) and *E. coli* TYR-14B1(p214C, pZBK-sts53-romt18) (C)

Reference

Chou, H.H., and Keasling, J.D. (2013). Programming adaptive control to evolve increased metabolite production. *Nat Commun* 4, 2595.

Hu, Y.L., Gai, Y., Yin, L., Wang, X.X., Feng, C.Y., Feng, L., et al. (2010). Crystal structures of a populus tomentosa 4-coumarate:Coa ligase shed light on its enzymatic mechanisms. *Plant Cell* 22(9)**,** 3093-3104.

Li, Z., and Nair, S.K. (2015). Structural basis for specificity and flexibility in a plant 4-coumarate:Coa ligase. *Structure* 23(11)**,** 2032-2042.

Robin, A.Y., Giustini, C., Graindorge, M., Matringe, M., and Dumas, R. (2016). Crystal structure of norcoclaurine-6-o-methyltransferase, a key rate-limiting step in the synthesis of benzylisoquinoline alkaloids. *Plant J* 87(6)**,** 641-653.

Wang, Y.C., Yi, H., Wang, M., Yu, O., and Jez, J.M. (2011). Structural and kinetic analysis of the unnatural fusion protein 4-coumaroyl-coa ligase::Stilbene synthase. *J Am Chem Soc* 133(51)**,** 20684-20687.
